# Optimizing Workplace Digital Mental Health Interventions: Systematic Review and Meta-Analysis

**DOI:** 10.2196/71253

**Published:** 2025-11-17

**Authors:** Elizabeth Stratton, Richard W Morris, Alyssa Milton, Mark Deady, Isabella Choi, Nick Glozier

**Affiliations:** 1 Faculty of Medicine and Health Central Clinical School University of Sydney Sydney, NSW Australia; 2 Centre of Excellence for Children and Families Over the Life Course Australian Research Council Sydney, null Australia; 3 Faculty of Medicine & Health, University of New South Wales Black Dog Institute Sydney, New South Wales Australia

**Keywords:** digital mental health interventions, workplace, employee, employee mental health, meta-analysis, systematic review

## Abstract

**Background:**

Digital mental health interventions (DMHIs) are widely used in workplaces to address common mental health conditions such as depression, anxiety, and stress. Their content and design vary widely, and meta-analyses repeatedly found substantial heterogeneity in their efficacy, but there is no systematic evaluation of the comparative effect of content on efficacy.

**Objective:**

We aimed to identify whether intervention design, therapeutic approaches, and content features are associated with greater efficacy in randomized controlled trials (RCTs) of DMHIs in workplaces.

**Methods:**

We conducted a systematic review of RCTs evaluating DMHIs using standardized measures of depression, anxiety, or stress in employed adults in 4 electronic databases (MEDLINE, PsycINFO, CENTRAL, and Embase) from 2004 to April 2024. A Bayesian multilevel meta-regression was used to estimate the pooled effect size (θ) and compare intervention characteristics (therapeutic approach, design, and content features) with sample demographics in explaining heterogeneity for each outcome. The influence of intervention characteristics on DMHI effectiveness was evaluated using posterior probabilities (pp) and evidence ratios (ERs), with ER>3 indicating likely therapeutic benefit. Risk of bias was assessed with the Cochrane Risk of Bias tool (version 2).

**Results:**

We included 81 RCTs evaluating 98 different DMHIs in 25,500 participants. Small but significant pooled effect sizes were identified for all 3 outcomes: depression (θ=0.167, 95% credible interval [CI] –0.31 to –0.03; ER 35.4; pp=0.972), anxiety (θ=–0.211, 95% CI –0.36 to –0.07; ER 113; pp=0.991), and stress (θ=–0.165, 95% CI –0.28 to –0.05; ER 199; pp=0.995), with intervention characteristics explaining more outcome heterogeneity than sample characteristics. In total, 71 (82.70%) trials had high risk bias, 4 (4.60%) had some concerns, and 11 (12.70%) had low risk bias, largely due to waitlist controls and inability to blind participants. Interventions using mindfulness and stress management approaches were more effective than cognitive behavioral therapy for anxiety and stress outcomes. Intervention designs incorporating person support (ERs 3.9-10.6) and expert design (stress: ER 25.7) enhanced therapeutic benefit for all outcomes. There was greater therapeutic benefit in interventions with video content (ER 3.69-5.71; pp=0.79-0.85) for all outcomes and those providing feedback scores (ER 6.55; pp=0.87), and reminder texts (ER 96.56; pp=0.99) showed moderate to strong benefit for stress outcomes.

**Conclusions:**

The effectiveness of workplace DMHIs in RCTs is influenced by their therapeutic approach, design, and content features. These influences can vary with the outcome targeted. Mindfulness and stress management approaches, expert involvement in design, and interventions blended with person support showed evidence of greater therapeutic benefit, as did some types of content. These findings inform a more deliberate, evidence-informed approach to addressing the lack of progress in improving DMHI efficacy in workplaces and highlight the limitations of assumptions of benefit from all features or participatory design processes without evaluation.

**Trial Registration:**

PROSPERO International Prospective Register of Systematic Reviews CRD42022323301; https://www.crd.york.ac.uk/PROSPERO/view/CRD42022323301

## Introduction

### Background

Digital mental health interventions (DMHIs) such as mobile apps, internet-based programs, and other technology-delivered initiatives have exploded onto the therapeutic landscape with estimates that 10-20,000 mental health apps currently exist [1-3]. Organizations have followed suit, and such interventions are widely implemented and promoted within workplace settings [4].

Emerging systematic review and meta-analytic evidence suggests that workplace-focused DMHIs that have been subject to trials produce small but significant reductions in symptoms of mental ill health among employees [4-7] and modest, potentially sustained increases in productivity and engagement in the workplace [8].

A critical finding from our 2022 meta-analyses is the significant heterogeneity observed between studies, with trial effect sizes ranging from –1.53 to +0.59 standardized mean differences [4]. This wide variability, including potential harm, underscores the need to understand the factors that influence intervention efficacy. Identifying these factors would enable developers to refine intervention designs and develop more targeted implementation strategies, with personalization of DMHIs seen as key [9].

While sample characteristics such as age, gender, and occupation, along with factors such as setting and context undoubtedly contribute to this outcome heterogeneity, and they are beyond the designer’s control. A systematic review [10] of smartphone apps for mental health found substantial variation in the content features, design, and implementation strategies. Characteristics such as the therapeutic approach, design of the intervention, and the content features are within the designer’s purview and could be optimized to enhance therapeutic effects.

### What Intervention Characteristics Might Influence DHMI Efficacy?

#### Therapeutic Approach

DMHIs vary in the therapeutic approach used in their design, but are often based on clinical models. Our previous review found that interventions with stress management and mindfulness-based approaches appeared more efficacious than cognitive behavioral therapy (CBT)–based approaches in managing symptoms of depression, anxiety, and stress [4]. Conversely, a meta-analysis by Karyotaki [11] found that internet-based CBT was more effective for depression than control treatment arms, though effect sizes varied across studies.

#### Design Approach

The involvement of mental health content experts, or people with lived experience, in the design and development of digital interventions may also be a significant factor contributing to the observed heterogeneity in effectiveness. The incorporation of person support, such as e-coach support to the end user, generally asynchronous, has been shown to enhance effectiveness in most, but not all systematic reviews [12].

#### Content Features

Meta-analyses have identified several key features and design elements that may influence the efficacy of DMHIs. Mood monitoring capabilities, which allow users to track their moods and symptoms, have been associated with greater reductions in depression and anxiety symptoms [13]. Additionally, apps that incorporate chatbot technology or conversational agents tend to show larger effects on mental health outcomes compared to those without this feature [13]. In terms of design, apps that incorporate more persuasive system design elements, such as personalization, reminders, and social support features, generally demonstrate greater efficacy in reducing symptoms likely due to increased engagement [14]. However, the relationship between features and user engagement is not always straightforward. Higher engagement does not necessarily translate to better mental health outcomes in all cases, highlighting the complex relationship between design, user interaction, and clinical efficacy [14].

The variability in reported outcomes across studies and reviews is likely due to a combination of statistical, methodological, and contextual factors, including heterogeneity in intervention design, study populations, outcome definitions, and analytic strategies. This variability may account for why, despite technological advancements, the effectiveness of these interventions for workplace mental health has not improved over nearly 2 decades [4].

However, prior meta-analyses—generally using frequentist approaches—have provided only partial insight into the complex array of factors influencing intervention effectiveness and have not focused on identifying key modifiable intervention aspects and their relative importance. Meta-regression techniques have often been used to identify how study-specific variables (usually termed moderators) explain study differences in efficacy. While a Bayesian framework enables the direct estimation of true study heterogeneity by incorporating prior knowledge, it also allows flexible estimation of moderator effects through hierarchical models, supporting comparative evaluation of different characteristics (such as those described earlier). This approach may enhance the interpretability of complex, multilevel data in ways that complement traditional meta-analytic techniques [15]. Bayesian hierarchical models can accommodate complex data structures and parse out moderating effects of study and intervention factors [16]. A multilevel Bayesian approach can also parse out the moderating effects of the study (sample and setting) from those of the intervention (therapeutic approach, design approach, and content features). Furthermore, Bayesian results, expressed as posterior probabilities (pp), provide an intuitive framework for decision-making in clinical practice [17].

This paper updates and extends a systematic review of the efficacy of workplace digital mental interventions to identify which intervention designs and features are associated with greater efficacy in randomized controlled trials (RCTs) through a Bayesian meta-regression. By doing so, we aim to provide valuable insights that can guide the development and implementation of more effective DMHIs in workplace settings.

## Methods

### Overview

This systematic review adhered to the PRISMA (Preferred Reporting Items for Systematic Reviews and Meta-Analyses) [18] guidelines (Multimedia Appendix 1) and was registered on PROSPERO (CRD42022323301). We aim to identify all published and unpublished, peer-reviewed, randomized controlled clinical trials of DMHIs targeted at employees that reported outcomes on a standardized mental health measure of depression, anxiety, or stress.

### Search Strategy

A systematic literature search was conducted in MEDLINE, PsycINFO, CENTRAL, and Embase from 2004 (when the first DMHI was identified) [7] until April 2024 using relevant keywords. Additionally, we manually searched selected journals and reference lists of included studies. Covidence systematic review software was used to manage the search, including automatic identification and removal of duplicates. The search strategy is provided in Multimedia Appendix 2. The search terms were developed from our previous systematic reviews [4,7].

### Study Selection Criteria

The selection criteria for the study are as follows:

- Participants: Working adults (aged 18-65 years) in paid employment.
- Interventions: DMHIs via websites, smartphones, tablets, or apps for employee mental health.
- Controls: Randomized control group (passive or active).
- Outcomes: Common mental disorder outcomes (depression, anxiety, and stress) as standardized mean differences from baseline to postintervention.
- Eligible measurements: Diagnostic interviews, professional diagnoses, or self-administered mental health scales.
- Excluded studies: Nonemployee-specific, nonworkplace, or in-person or telephone or email-only interventions and non-English language publications.

### Identification of Studies

Two authors (ES and IC) independently screened titles, abstracts, and full texts. Discrepancies were resolved through consensus with the senior author (NG).

### Data Extraction and Coding

#### Overview

We extracted the mean and SD of distress, depression, and anxiety and sample size (n) in each arm (intervention and control) of each study at baseline and first posttreatment follow-up (first date of data extraction June 3, 2024). Data extraction was completed independently by 2 authors (ES and IC) and verified by NG. All data were entered into a structured Microsoft Excel spreadsheet for consistency, with coding of intervention and sample characteristics cross-checked by the review team.

Sample characteristics include age and gender distribution, country, and industry sector.

#### Intervention Characteristics (Meta-Regression Moderators)

##### Therapeutic Approach

The therapeutic approach of the intervention was taken as described by the authors. If not specified, the content and examples in the paper were categorized by the reviewers, if possible: CBT, stress management (no specific cognitive or behavioral techniques identified), mindfulness (including other third-wave approaches), and other—coded when the approach described did not fit into the 3 approaches mentioned (eg, decision aid tools and mental contrasting).

##### Design Approach

The design approach was categorized as described by the authors:

- Designed primarily by mental health experts (eg, clinical psychologists, social workers, and psychiatrists), bachelor- or master-level students (eg, psychology, clinical psychology, and counseling), and other trained therapists.
- Co-design with people with lived experience: Report the involvement of people with a lived experience of depression, anxiety, or stress-related disorder (in either the registered protocol or original report) during the development of the intervention.
- Person support: The provision of support to the end user from online professionals such as mental health clinicians. Technical support only was not considered person support. Interventions and studies using adjunctive clinical face-to-face interventions were excluded.

##### Intervention Features

Intervention features as identified in the papers were incorporated in the following categories: audio narration, avatar, discussion forums or testimonials, feedback scores, homework or diary, mood tracker, participants can select module content, reminder emails, reminder texts, tailored for symptoms, and videos. Authors were contacted if sufficient details were not reported.

### Quality Assessment and Risk of Bias Within Studies

Risk of bias was assessed using the Cochrane Risk of Bias tool (version 2) [19]. Two authors (ES and IC) independently evaluated 5 domains: randomization, intervention deviations, missing data, outcome measurement, and result selection. Studies were categorized as “low risk,” “some concerns,” or “high risk” based on these assessments. Multiple intervention arms were evaluated individually.

### Statistical Analysis

Data were synthesized using a Bayesian multilevel meta-regression model. This approach accounted for the nested structure of the data (interventions within studies) and enabled estimation of pooled effect sizes, comparison of effects, and assessment of moderator impacts, all with accompanying credible intervals (CIs). Posterior distributions were used to interpret the strength of evidence, with evidence ratios (ER>3) indicating likely therapeutic benefit. Full model specifications and synthesis logic are provided in Multimedia Appendix 3.

All analyses were undertaken using R (version 4.2.0; R Foundation for Statistical Computing). Meta-regressions were performed using brms (version 2.17.0), an R interface to Stan (version 2.21.5).

We summarized the included studies, describing sample and intervention characteristics both overall and for each specific outcome. Pooled effect sizes (compared to placebo) for the efficacy of interventions for each specified outcome and their heterogeneity were estimated using a multilevel approach, as outlined by Harrer et al [20].

The meta-regression model was defined as:



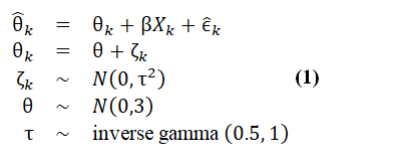



In this model, 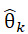 represents the observed effect size of each study; θ denotes the true pooled effect size when all other moderators equal 0; β is a vector of model coefficients; *X_k_* is a matrix of moderators and baseline differences; 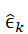 represents the within-study sampling error, which is modeled as a normally distributed random variable with known variance σ^2^*ₖ* derived from study-level data; and ζ*_k_* represents true between-study heterogeneity with variance τ^2^.

To explore the factors potentially influencing effectiveness, we conducted a moderator analysis. We calculated the proportion of between-study variance explained by moderators using the formula:



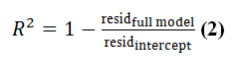



where the resid_intercept_ represents the residual between-study variance in the meta-analysis model, which included the intercept and baseline group difference only (Multimedia Appendix 3). The full model included the intercept and two sets of moderators: (1) baseline study sample characteristics and (2) intervention characteristics. Thus, *R*^2^ represents the proportion of between-study heterogeneity explained by each set of moderators (sample vs therapeutic approach vs design vs content features).

We estimated the individual effect of each intervention characteristic on symptom reduction for each of the intervention characteristics (grouped by therapeutic approach, design approach, and intervention features). For each β, we determined the effect on symptom reduction, marginalizing over all other intervention characteristics. The average marginal effect of each characteristic (β) is presented to indicate the size and direction of the effect size. We also provided the 0.025 and 0.975 quantiles containing 95% of the posterior values as a 95% CI of the range of likely effects. Related to this is the pp of a “therapeutic effect,” that is, *P*(β<0) (*pp*), where a value over 0.95 arbitrarily indicates that specific moderator is highly likely to have a marginal therapeutic benefit (ie, distinct from a *P*<.05). Finally, the ratio of a therapeutic benefit over harm (benefit/harm; *P*(β*_p_*<0)/*P*(β*_p_*>0)) was quantified using the Bayesian ER as described by Kass and Raftery [21]. We interpreted ER>3 as indicating moderate evidence and ER>10 as strong evidence for a nonzero therapeutic effect, relative to the null. ERs are not interpreted as direct probabilities or odds, but rather as a relative support for the model including an effect compared to the model without an effect.

### Sensitivity Analyses

To evaluate robustness to prior specification, we conducted sensitivity analyses using alternative τ priors with wider and narrower distributions: inverse_gamma(0.25, 1), inverse_gamma(0.10, 1), and inverse_gamma(0.50, 2), in addition to the primary inverse_gamma(0.50, 1) prior. In the inverse gamma prior distributions, the shape (α) and scale (β) parameters are reported explicitly to avoid duplication of Greek symbols used elsewhere in the model. The shape parameter controls the peak density and tail weight of the distribution, while the scale parameter determines its overall spread. For each specification, we examined changes in the posterior mean and 95% CI of τ and in the posterior distributions of the regression coefficients (β).

## Results

### Search Results

**Figure 1:**
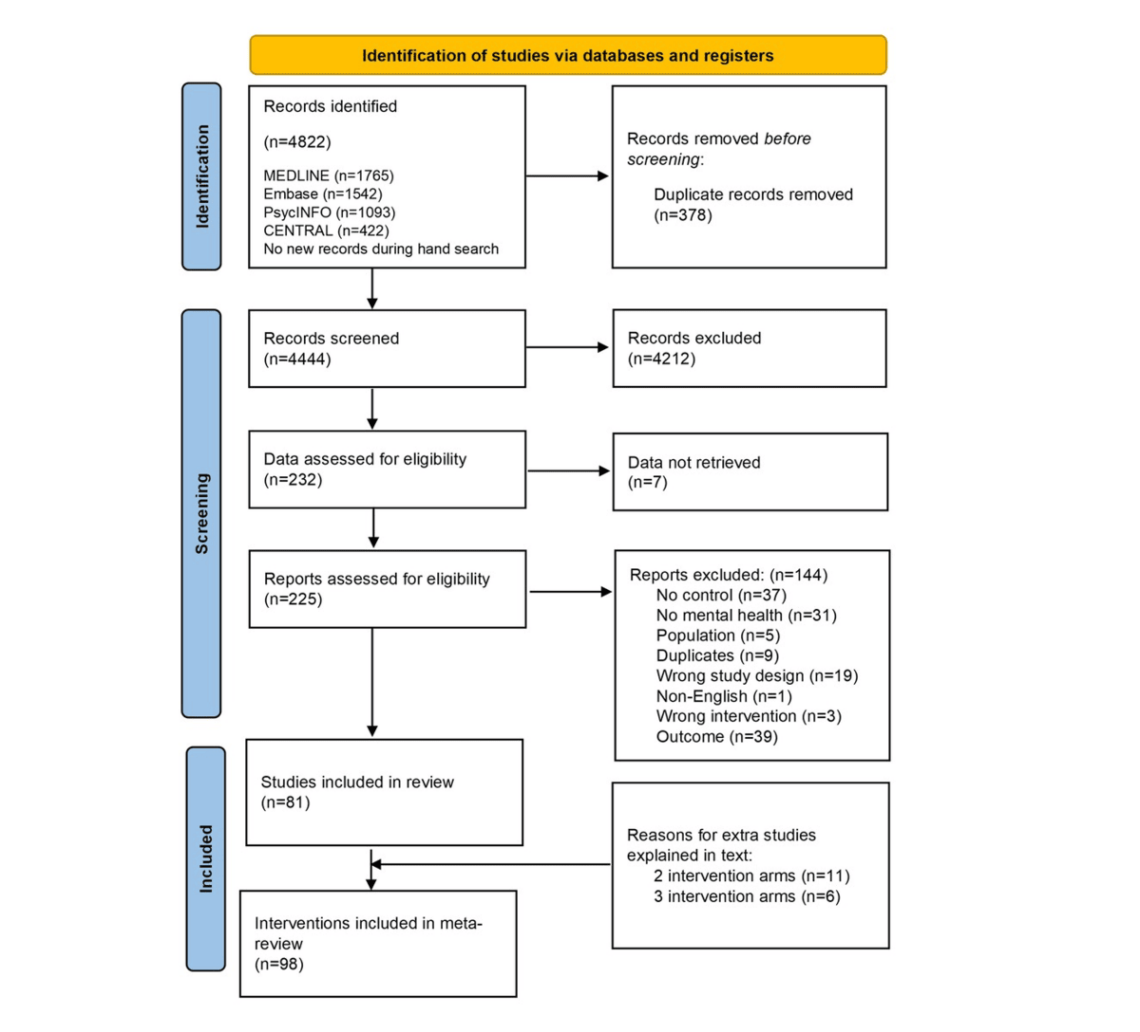
Flow diagram of studies.

The search identified 4822 titles (Figure 1), and no additional studies were identified through manual searches. After removing duplicates, a total of 4444 titles and abstracts were reviewed (ES and IC). In total, 4212 papers were excluded based on eligibility criteria, leaving 232 for full-text review. Seven authors did not provide the requested data, and 144 papers were excluded for various reasons (Figure 1). In total, 81 RCT studies met eligibility criteria and were retained for analysis [22-102]. Studies with multiple intervention arms were treated as individual interventions, resulting in 98 total interventions for analysis.

### Risk of Bias

Of the included studies, 11 (12.7%) [31,33,35,42,58,84,93,95,101-103] had low bias risk, 4 (4.6%) [38,71,91,104] had some concerns, and 71 (82.7%) [16, 23-30, 32, 34, 36, 37, 39, 40, 43-57, 59-66, 68-70, 72-83, 85-90, 92, 94, 96-100, 105-109] had high bias risk. Main issues were deviations from intended interventions and missing outcome data, common in DMHI research. Notably, 50 (58%) were rated high risk due to using waitlist control groups, preventing user blinding (Multimedia Appendix 4) [16,23-40,42-66,68-109].

A visual summary of risk of bias assessments is presented in Multimedia Appendix 4, following the standard color convention of the Risk of Bias tool (version 2): green (low risk), yellow (some concerns), and red (high risk). The figure summarizes the 5 core bias domains for each included study.

### Summary of Overall Interventions Identified

We identified 81 RCT studies, evaluating 98 different interventions in 25,480 participants across various industries, including health care, insurance, management, information technology, telecommunications, education, government, marketing, retail, banking, and human resources. The studies were conducted in 18 countries: the United States (n=14, 17.28%), followed by Germany (n=13, 16.05%) and Japan (n=13, 16.05%). The United Kingdom contributed 10 (12.35%), while both Sweden and Australia accounted for 7 (8.64%) each. There were 5 (6.17%) studies from the Netherlands. A smaller number of studies came from Italy (n=2, 2.47%), Hong Kong (n=2, 2.47%), and broader Europe (n=2, 2.47%), as well as from Korea (n=1, 1.23%). Single studies were reported from Finland (n=1, 1.23%), China (n=1, 1.23%), Brazil (n=1, 1.23%), Singapore (n=1, 1.23%), and a combination of the United States and Canada (n=1, 1.23%).

Weighted mean ages were 41-42 (range 22.8-57.5) years, with female participation averaging from 55% to 60.6% (range 4.9%-100%). Multimedia Appendix 5 shows the distribution of intervention characteristics for each outcome, with “N” indicating the number of interventions per characteristic used for each outcome.

The most common therapeutic approach was CBT for stress, depression, and anxiety. Most interventions were designed by clinical experts or in consultation with clinical experts, and almost half included person support. There were proportionately fewer studies with lived experience co-design (stress: n=8, 11%; depression: n=10, 17%; and anxiety: n=5, 12%). The most commonly reported features were audio narration (stress: 51, 72%; depression: n=44, 75%; and anxiety: n=34, 83%), and a homework diary (stress: n=54, 76%; depression: n=44, 75%; and anxiety: n=30, 73%); however, few were tailored for symptoms (stress: n=2, 3%; depression: n=4, 7%; and anxiety: n=4, 10%).

### Symptom-Specific Therapeutic Benefits of DMHIs

#### Overview

Therapeutic benefit is indicated by a reduction in symptoms of stress, anxiety, or depression; therefore, negative estimates represent improvement or benefit. Using a multilevel model, which included a random intercept for the individual studies, we estimated the therapeutic effect of all interventions combined adjusted for baseline differences (but without inclusion of the intervention characteristics), reaffirming strong and highly probable evidence of benefits [4]:



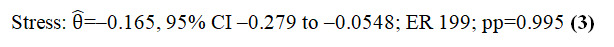





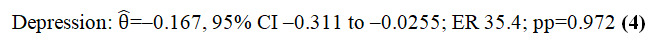





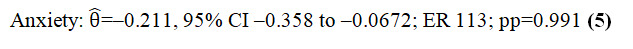



The ER exceeded 20 (indicating strong evidence of benefit), and the pp surpassed the 95% credibility threshold for each outcome.

#### Heterogeneity of Outcomes Across Studies

**Figure 2:**
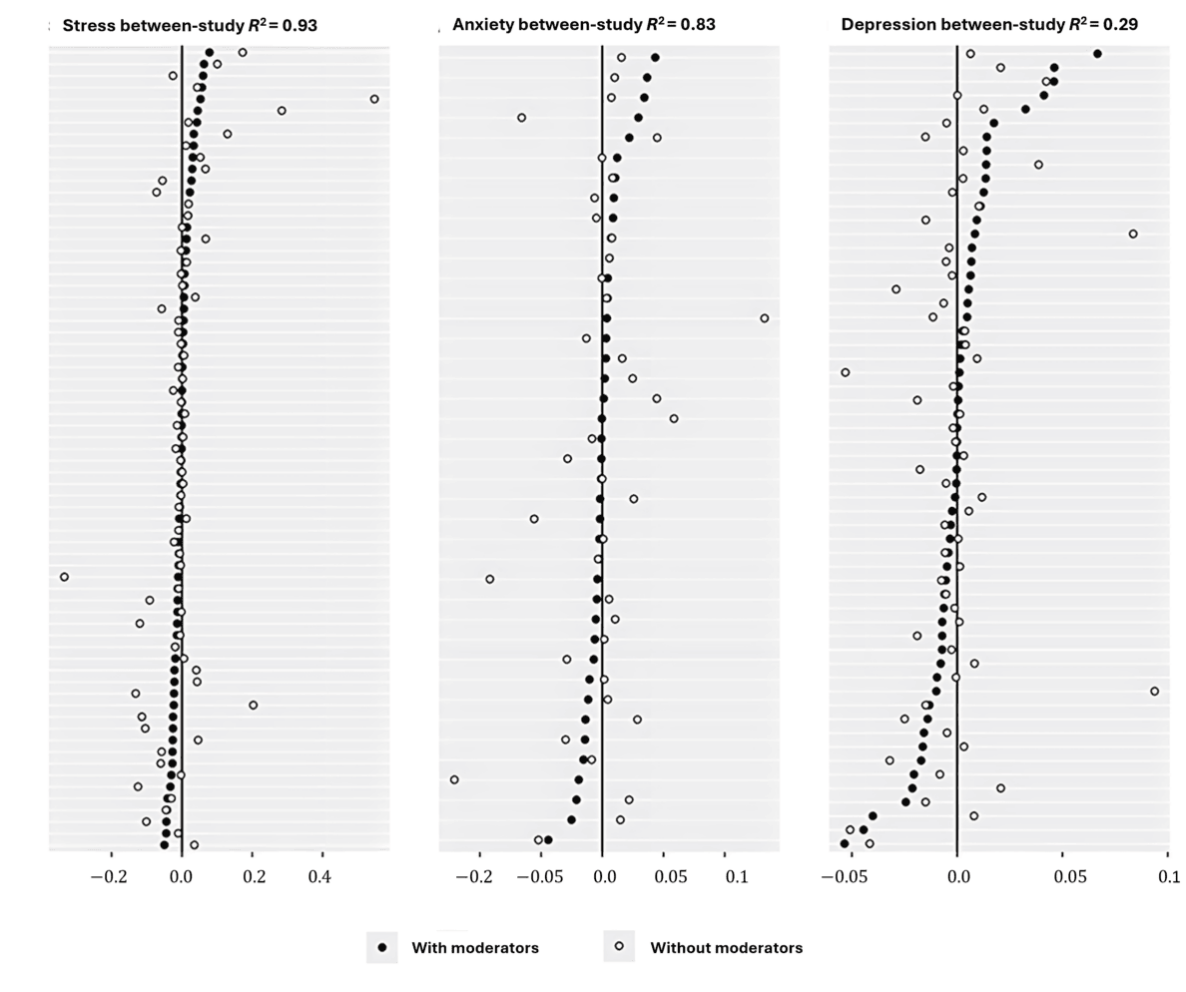
Impact of sample and design moderators on between-study variance for each.

The estimates of heterogeneity (τ^2^) from the models were all above 0 and differed only slightly for each outcome. The τ estimates for stress, depression, and anxiety treatment effects were 0.28 (95% CI 0.19-0.40), 0.20 (95% CI 0.11-0.34), and 0.23 (95% CI 0.12-0.38), respectively. The 95% CIs excluded 0, indicating the presence of heterogenous treatment effects. We examined the proportion of between-study variance explained by the addition of the 2 groups of moderators in the meta-regressions below. Figure 2 and Table 1 illustrate the between-study variance among treatment effects reported, as revealed by our meta-analysis, with and without accounting for the impact of the potential moderating variables.

**Table 1:** Proportion of variance explained (R2) of each outcome by sample and intervention characteristics^a^.

Source	Sample characteristics, *R*^2^	Therapeutic and design approach, *R*^2^	Intervention features, *R*^2^	All intervention characteristics, *R*^2^	Sample and intervention characteristics combined, *R*^2^
Stress	0.21	0.67	0.64	0.82	0.93
Depression	0.27	0.38	0.32	0.22	0.29
Anxiety	0.55	0.55	0.42	0.67	0.83

^a^The variances do not add to 1 because they are individual models.

#### Sensitivity to Prior Specification

Posterior τ estimates were stable across all alternative priors (Multimedia Appendices 6 and 7). For example, for stress, the τ posterior mean ranged from 0.28 (95% CI 0.19-0.40) under the primary prior to 0.31 (95% CI 0.22-0.44) under the widest prior (inverse_gamma(0.50, 2)), with overlapping 95% CIs. The largest shift occurred when β was increased from 1 to 2, but this did not alter substantive conclusions. Regression coefficient estimates (β) showed minimal change across prior specifications (Multimedia Appendix 7).

Figure 2 visually compares the study-specific treatment effects from 2 analyses. The empty circles represent effects from the meta-analysis without sample or intervention characteristics, while the filled circles show effects from the meta-regression including a combination of all sample and intervention characteristics. We observe that the empty circles typically fall further from the population treatment effect (represented as 0 on the x-axis) compared to the filled circles.

We aimed to determine the proportion of heterogeneity explained by moderating sample and intervention characteristics (Table 1). When stress was the outcome, we find that sample characteristics explain only 21% of the variance, while the intervention characteristics of design and therapeutic approach and intervention features account for 67% and 64%, respectively, when assessed independently and explain 82% of the variance together. When combined, the sample and intervention moderators explained 93% of the between-study variance in the efficacy of studies with stress as the outcome.

The sample and intervention moderators explained much less of the variance when depression was the outcome. For depression interventions, we observe a different pattern. Sample characteristics explain only 27% of the variance, design approach accounting for 38%, and intervention features for 32%. The combination of all intervention characteristics only explains 22% of the variance, while all characteristics combined together account for less than one-third of the variance (29%).

With anxiety as the outcome, sample characteristics explained 55% of the variance, design approach 55%, and intervention features 32%. The combination of designs and features explained 67% of the variance, while all moderators together account for 83%.

### Comparative Impact of Intervention Characteristics on Therapeutic Benefit

#### Therapeutic Approaches

Mindfulness-based approaches showed greater therapeutic benefit than CBT for all outcomes. Stress management approach–based interventions showed greater therapeutic benefits than CBT for depression and stress but not anxiety. Specifically, stress management showed a moderate benefit than CBT (ER 9.8) for stress reduction outcomes. Mindfulness showed moderate evidence of benefit compared to CBT (ER 3.0) for depression and a moderate benefit (ER 3.6) for anxiety. The evidence did not distinguish between the benefits of mindfulness and stress management for any outcome (see therapeutic approach in Table 2).

**Table 2:** Comparative impact of individual intervention moderators on psychological improvement—therapeutic approach, design approach, and intervention features.

Outcome				β (95% CI^a^)		pp^b^		ER^c^
**Therapeutic approach**								
	**Stress**							
		Mindfulness>CBT^d^	–.20 (–0.73 to 0.33)		0.73		2.80	
		Mindfulness>stress management	.12 (–0.48 to 0.71)		0.37		0.60	
		Stress management>CBT	–.32 (–0.73 to 0.08)		0.91		9.80^e^	
	**Depression**							
		Mindfulness>CBT	–.30 (–1.00 to 0.43)		0.75		3.00^e^	
		Mindfulness>stress management	–.14 (–1.00 to 0.71)		0.60		1.50	
		Stress management>CBT	–.16 (–0.70 to 0.39)		0.68		2.10	
	**Anxiety**							
		Mindfulness>CBT	–.35 (–1.10 to 0.36)		0.78		3.60^e^	
		Mindfulness>stress management	–.25 (–1.10 to 0.59)		0.68		2.20	
		Stress management>CBT	–.10 (–0.62 to 0.43)		0.63		1.70	
**Design approach**								
	**Stress**							
		Expert design, yes or no	–.32 (–0.59 to –0.03)		0.96		25.67^e^	
		Lived experience design, yes or no	.21 (–0.06 to 0.50)		0.09		0.10	
		Person support, yes or no	–.12 (–0.37 to 0.14)		0.80		3.96^e^	
	**Depression**							
		Expert design, yes or no	.07 (–0.32 to 0.46)		0.38		0.61	
		Lived experience design, yes or no	.01 (–0.27 to 0.30)		0.47		0.90	
		Person support, yes or no	–.23 (–0.60 to 0.14)		0.85		5.79^e^	
	**Anxiety**							
		Expert design, yes or no	–.05 (–0.69 to 0.57)		0.55		1.23	
		Lived experience design, yes or no	.10 (–0.27 to 0.49)		0.33		0.50	
		Person support, yes or no	–.27 (–0.60 to 0.05)		0.91		10.56^e^	
**Intervention features**								
	**Stress**							
		Audio narrator, yes or no	–.08 (–0.34 to 0.17)		0.69		2.23	
		Avatar, yes or no	–.04 (–0.48 to 0.39)		0.55		1.24	
		Discussion forum testimonial, yes or no	–.19 (–0.52 to 0.16)		0.83		4.76^e^	
		Feedback scores, yes or no	–.17 (–0.42 to 0.08)		0.87		6.55^e^	
		Homework diary, yes or no	–.24 (–0.48 to 0.00)		0.95		19.00^e^	
		Mood tracker, yes or no	.10 (–0.16 to 0.35)		0.27		0.36	
		Participants can select content, yes or no	–.18 (–0.81 to 0.45)		0.69		2.22	
		Reminder emails, yes or no	–.01 (–0.26 to 0.23)		0.54		1.16	
		Reminder texts, yes or no	–.39 (–0.67 to –0.10)		0.99		96.56^e^	
		Tailored for symptoms, yes or no	–.06 (–0.87 to 0.78)		0.55		1.21	
		Videos, yes or no	–.11 (–0.36 to 0.13)		0.79		3.69^e^	
	**Depression**							
		Audio narrator, yes or no	–.11 (–0.43 to 0.20)		0.72		2.57	
		Avatar, yes or no	.04 (–0.27 to 0.36)		0.41		0.71	
		Discussion forum testimonial, yes or no	.13 (–0.18 to 0.44)		0.24		0.32	
		Feedback scores, yes or no	.06 (–0.25 to 0.37)		0.38		0.62	
		Homework diary, yes or no	–.03 (–0.33 to 0.25)		0.57		1.32	
		Mood tracker, yes or no	–.01 (–0.29 to 0.29)		0.54		1.17	
		Participants can select content, yes or no	.07 (–0.23 to 0.37)		0.34		0.51	
		Reminder emails, yes or no	–.10 (–0.38 to 0.18)		0.72		2.60	
		Reminder texts, yes or no	.03 (–0.26 to 0.33)		0.43		0.76	
		Tailored for symptoms, yes or no	.14 (–0.16 to 0.47)		0.23		0.30	
		Videos, yes or no	–.18 (–0.46 to 0.11)		0.85		5.71^e^	
	**Anxiety**							
		Audio narrator, yes or no	–.16 (–0.61 to 0.28)		0.73		2.77	
		Avatar, yes or no	.06 (–0.30 to 0.46)		0.40		0.65	
		Discussion forum testimonial, yes or no	.05 (–0.28 to 0.37)		0.41		0.68	
		Feedback scores, yes or no	–.17 (–0.50 to 0.15)		0.80		4.07^e^	
		Homework diary, yes or no	–.12 (–0.51 to 0.25)		0.70		2.34	
		Mood tracker, yes or no	.13 (–0.21 to 0.49)		0.27		0.36	
		Participants can select content, yes or no	.11 (–0.24 to 0.47)		0.30		0.43	
		Reminder emails, yes or no	–.11 (–0.45 to 0.23)		0.69		2.22	
		Reminder texts, yes or no	–.13 (–0.45 to 0.19)		0.74		2.91	
		Tailored for symptoms, yes or no	.15 (–0.18 to 0.47)		0.22		0.28	
		Videos, yes or no	–.16 (–0.48 to 0.15)		0.81		4.30^e^	

^a^CI: credible interval.

^b^pp: posterior probability.

^c^ER: evidence ratio.

^d^CBT: cognitive behavioral therapy.

^e^Probable positive evidence.

#### Design Approach

The intervention’s design approach had varying influence on effectiveness across stress, depression, and anxiety outcomes. For instance, interventions incorporating person support were consistently associated with moderate to strong benefits across all 3 outcomes. Person support was associated with moderate to strong evidence of benefit, with an ER of 3.96 for stress, 5.79 for depression, and 10.56 for anxiety. Further, interventions that explicitly acknowledged expert design showed strong evidence of greater benefit for stress reduction (ER 25.67). However, it did not demonstrate significant benefits for depression or anxiety outcomes. Co-design involving individuals with lived experience did not produce greater benefits for any outcome compared to interventions without such co-design. ERs were below 1 for all 3 conditions (stress, depression, and anxiety), suggesting no additional benefit from this approach (see design approach in Table 2).

#### Intervention Features

The intervention’s features had varying influence on effectiveness across stress, depression, and anxiety outcomes. Notably, videos showed moderate evidence of benefit across all 3 outcomes, with ERs ranging from 3.7 to 5.7. Evidence was strongest for depression (ER 5.71) and anxiety (ER 4.30). Reminder texts demonstrated strong evidence of benefit for stress reduction (ER 96.56) and moderate evidence for anxiety. Homework diaries also showed strong evidence of benefit for stress (ER 19.00). Feedback scores were associated with moderate evidence of benefit for both stress (ER 6.55) and anxiety (ER 4.07). Discussion forum testimonials demonstrated moderate evidence of benefit for stress (ER 4.76). Some features, such as mood trackers, avatars, and participant content selection, showed no evidence of benefit across all outcomes (see intervention features in Table 2).

## Discussion

### Principal Findings

This comprehensive study used a Bayesian approach to a systematic review of 81 RCTs encompassing 98 distinct DMHIs for stress, depression, and anxiety in the workplace. We evaluated the influence of study and intervention moderators on the heterogeneity and therapeutic effect observed in these studies. In this updated review, we confirmed previous findings that DMHIs yield small but positive effects in alleviating symptoms of mental ill health among employees in occupational settings [4-7].

Our analysis of between-study variance revealed intriguing patterns across different mental health outcomes. For stress interventions, we found that intervention characteristics explained a substantial proportion of the variance (82% combined), while sample characteristics account for only 21%. This suggests that for stress, the design and features of digital interventions play a more crucial role in determining the effectiveness than the study sample. Anxiety outcomes show a more balanced picture, with sample characteristics explaining 55% of the variance and intervention characteristics accounting for 67% when combined. This indicates that both who uses the intervention and how it is designed are important factors in anxiety treatment outcomes. Conversely, in studies where depression was the outcome, neither sample characteristics (27%) nor intervention characteristics (22% combined) explain a large proportion of the variance. This finding suggests that other unidentified factors may be playing a significant role in determining effectiveness of digital interventions for depression. Our previous review [4] showed that between-study heterogeneity in depression outcomes was lower, with only moderate levels of heterogeneity observed compared to high levels in both anxiety and stress. In this study, we observed a similar trend, where the observed variance between studies was lower in depression compared to stress and anxiety, suggesting that there may actually be less variation to account for in interventions focusing on depression.

The relatively low impact of sample characteristics on variance in stress and depression interventions is particularly noteworthy, challenging assumptions about the primacy of individual factors in treatment outcomes. Our findings validate the need for further exploration of intervention characteristics, especially for stress and anxiety interventions, where they explain a substantial portion of the variance.

### Therapeutic Focus of Digital Workplace Mental Health Interventions

The meta-regression analysis provided additional insights into the impact of various intervention characteristics on treatment efficacy. Notably, mindfulness-based approaches demonstrated the highest probability of therapeutic benefit for employees experiencing depression and anxiety, while stress management techniques showed the greatest promise for those dealing with stress. These findings highlight the importance of tailoring intervention approaches to these specific mental health concerns. Interestingly, while DMHIs have shown effectiveness in reducing stress symptoms, their efficacy in treating depression appears more nuanced [4]. Contrary to expectations, the incorporation of the well-established face-to-face clinical approach CBT, when delivered via DMHI platforms, showed a lower probability of benefit for depression compared to other methods.

The mixed results for depression treatment via DMHIs underscore the complex nature of this condition. Depression often requires more intensive personalized care that may be challenging to deliver solely through digital platforms [110]. For instance, digital mindfulness-based interventions for depression face challenges in providing personalized care [111] and replicating the human connection [112] found in face-to-face settings. These limitations may impact their effectiveness in addressing the complex, fluctuating, and nuanced needs of individuals with depression, who often benefit from real-time adjustments and supportive interpersonal dynamics. Drawing on decades of research and experience, hybrid solutions that combine face-to-face and digital treatments are likely to be the most effective, offering a balanced approach that can address diverse needs [113,114].

### Co-Designing Digital Workplace Mental Health Interventions

Explicit involvement of mental health experts in the intervention design was associated with a higher probability of benefit for stress reduction, aligning with research highlighting the value of expert involvement in enhancing the effectiveness and safety of mental health interventions [115]. However, such involvement did not lead to great benefit for depression and anxiety. While digital interventions offer promising avenues for mental health support, the depth and complexity of depression and anxiety may necessitate more comprehensive approaches outside of what experts may rely on in clinical care.

DMHIs co-designed by individuals with personal experience of mental health conditions did not demonstrate greater therapeutic benefit than those not co-designed for any outcome. The null effect of lived experience co-design may be attributed to the lack of current studies, with lived experience added to the design approach in a mere 10% of the studies but warrants more exploration, given (1) its emerging evidence in adjacent DMHI fields [116] and (2) further confirmation or rejection of these null findings. A final caveat to these findings may be that the ascertainment involvement of experts and people with lived experience depended on explicit mention in the papers and may have been misclassified.

As with most other meta-analyses and now meta-reviews [12,117], our results confirmed that the provision of adjunctive support had a high probability of increasing benefit across all 3 mental health outcomes: stress, depression, and anxiety. This finding underscores the importance of human support in the form of an e-coach for delivery of digital interventions, suggesting that a blended approach combining technology with personal guidance may be most effective [113,114].

### Effective Features of Digital Workplace Mental Health Interventions

A novel result from this study was the identification of potentially more efficacious features. Using as accepted ER>3 as indicative of useful features, we found that video use appeared beneficial transdiagnostically and that providing individuals with the mental health feedback scores leads to probable treatment benefits for the reduction of stress and anxiety. This aligns with the hypothesis that such feedback can increase awareness of one’s condition and motivate engagement in treatment [118]. By recognizing the severity of their symptoms, individuals may be more likely to adhere to treatment, potentially leading to improved outcomes [119]. Videos prove effective in delivering psychoeducation and support, making complex information more accessible through visual and auditory content, potentially improving understanding and retention [120]. Research by Berry et al [120] found video-based interventions particularly engaging and appealing to participants.

There were no other features that consistently stood out as beneficial, although there were strong effects for homework diaries and reminder texts for stress outcomes. Homework diaries and reminder texts features may help maintain user engagement and motivation throughout the intervention process, although we cannot explain why they should have an effect on stress but not depression and anxiety. It may be that such features have mixed effects in a study on novel digital mental health assessment tool, which revealed generally positive perceptions of reminders among users [121]. However, the same study also identified a potential drawback: some users found frequent reminders intrusive, suggesting the need for careful implementation. In the same manner, a review on supporting homework compliance in CBT apps suggested that homework can support in-the-moment self-assessment reflections. However, the review also noted that feedback on the inconvenience of doing homework may hamper a patient’s motivation [122]. These contrasting findings underscore the importance of thoughtful design in DMHIs. Future research should consider the optimal delivery of reminders, for instance, the frequency and timing of such reminders, as well as ways to make homework features engaging and convenient for users. Striking the right balance between effective intervention and user experience will be crucial for maximizing the benefits of digital mental health tools.

The study’s findings on mood trackers present an unexpected contrast to their widespread use in DMHIs. Despite being used in about 20% of the reviewed studies and often preferred by users [123], mood trackers showed a low probability of benefit and potentially detrimental impacts on anxiety and stress. This outcome is surprising, given their demonstrated effectiveness in previous RCT [124]. Several hypotheses could explain this result. Constant mood monitoring might lead individuals to focus excessively on negative emotions, potentially exacerbating symptoms. Additionally, the pressure to accurately track and report mood could create a sense of burden, contributing to increased stress and reduced intervention adherence. These findings highlight the complexity of designing effective DMHIs. Features that are intuitively appealing or unappealing may not always yield positive outcomes. Moving forward, developers and researchers may need to reassess the implementation of mood trackers in DMHIs, exploring ways to mitigate potential negative effects or considering alternative methods for monitoring emotional well-being.

### Limitations

This study offers valuable insights into improving workplace DMHIs for mental health, but it is crucial to acknowledge its limitations. Most included studies had a high risk of bias, potentially affecting the reliability of the findings. The high heterogeneity among studies might also have influenced results. Another key limitation is the use of the Bayesian approach, which only allows for estimating the probability of benefits rather than standardized effects. This means we can identify which features are likely beneficial but cannot quantify the exact magnitude of these benefits across different interventions and outcomes. The proportion of variance explained by the current set of moderators was substantially lower for depression (*R*^2^=0.29) compared to stress (*R*^2^=0.93) and anxiety (*R*^2^=0.83). This suggests that the factors influencing intervention effectiveness for depression may differ or be more complex and that the current moderators—largely focused on intervention type and features—may be insufficient for explaining variation in depression outcomes. Future work may benefit from exploring additional depression-specific moderators such as symptom severity, intervention dosage, or therapeutic intensity to better model these effects. This study is also limited by including only studies published in the English language.

### Conclusions

The study found small but positive effects on employee mental health, with intervention characteristics explaining more variance than sample demographics, particularly for stress interventions. Mindfulness-based approaches were most effective for depression and anxiety, while stress management techniques excelled in stress reduction. The analysis emphasizes the importance of co-designing interventions with mental health experts and incorporating adjunctive support to enhance effectiveness. It also highlights the need for thoughtful implementation of features like reminders and homework as well as a critical reassessment of mood trackers, which showed potentially detrimental effects despite their popularity.

These findings emphasize the need for a more systematic approach to DMHI design, considering factors such as the specific mental health outcome, integration of professional expertise, balance of automated features and human support, and optimal implementation of engagement tools. Future research should focus on developing a comprehensive framework to guide the design, implementation, and evaluation of digital workplace mental health interventions.

## Appendix

Multimedia Appendix 1 PRISMA checklist.

Multimedia Appendix 2 Search terms.

Multimedia Appendix 3 Model details (from model definition).

Multimedia Appendix 4 Estimated risk of bias across all studies.

Multimedia Appendix 5 Distribution of intervention characteristics, therapeutic and design approaches, and intervention features used in workplace digital mental health intervention.

Multimedia Appendix 6 τ posterior means (±95% CI) from each prior.

Multimedia Appendix 7 (A) Probability density functions and (B) posterior β values from each prior.

## Abbreviations

CBT: cognitive behavioral therapy
CI: credible interval
DMHI: digital mental health intervention
ER: evidence ratio
pp: posterior probability
PRISMA: Preferred Reporting Items for Systematic Reviews and Meta-Analyses
RCT: randomized controlled trial
