# Supplementary material for: Optimizing Workplace Digital Mental Health Interventions: Systematic Review and Meta-Analysis
Source: J Med Internet Res. 2025 Nov 17;27:e71253. doi: 10.2196/71253 (PMC12670063; doi:10.2196/71253)
Supplement: Multimedia Appendix 2 [file jmir_v27i1e71253_app2.docx]

Search criteria:

Databases Searched: MEDLINE (via PubMed), PsycINFO (via Ovid), Cochrane CENTRAL, EMBASE (via Elsevier)

| Description PubMed (MEDLINE) | AND OR method |
| --- | --- |
| Mental health terms | stress* [Tittle/Abstract] OR mental health [Title] OR mental illness [Title] OR mental disorder* [Title] OR depress* [tw] OR anxi* [tw] OR affective symptom* [sh] mood disorder*. tw OR mental health condition* [tw] OR mental health [sh] mental ill* [tw] psychological.tw |
| Work context terms | Employment[Mesh:NoExp] OR Occupational Groups[Mesh] OR Professional[Mesh] OR Occupations[Mesh] OR Workplace[Mesh] OR Workload[Mesh] Employment, Supported (Mesh) Health Workforce (Mesh) |
|  | work*[Title] OR occupation*[Title] OR employ*[Title] OR job*[Title] OR professional*[Title] OR organi?ational*[Title] OR workplace*[tw] orker*.tw, manager*.tw work place*.tw, supervisor*.tw |
| Search for DMHI | ("digital"[Title/Abstract] OR "online"[Title/Abstract] OR "web-based"[Title/Abstract] OR "internet"[Title/Abstract] OR "eHealth"[Title/Abstract] OR "mHealth"[Title/Abstract] OR "mobile app"[Title/Abstract] OR "smartphone"[Title/Abstract] OR "computerized"[Title/Abstract] OR "teletherapy"[Title/Abstract] OR "virtual"[Title/Abstract] OR "technology-based"[Title/Abstract]) |
| Search for interventions | prevent* [tw] OR intervention* [tw] stress manag* [tw] OR program* [tw] OR "cognitive behavioral therapy" OR CBT OR "CBT-based" OR "internet-based CBT" OR "mindfulness" OR "mindfulness-based" OR "MBCT" OR "MBSR" OR "stress management" OR "stress reduction" OR "relaxation techniques" OR "relaxation therapy" OR "positive psychology" OR "self-guided therapy" OR "self-help" OR "problem-solving therapy" OR "acceptance and commitment therapy" OR ACT OR "dialectical behavior therapy" OR DBT OR "behavioral activation" OR "third-wave therapy" OR "mental contrasting" OR "resilience training" OR "emotion regulation training" OR "psychoeducation" |
| Study type | (("randomized controlled trial"[Publication Type] OR "controlled clinical trial"[Publication Type] OR randomized[Title/Abstract] OR randomised[Title/Abstract] OR RCT[Title/Abstract]) |
| Limited | (english language and humans and "all adult (19 plus years)") |

| Description PsychINFO | AND OR method |
| --- | --- |
| Mental health terms | stress* [Tittle/Abstract] OR mental health [Title] OR mental illness [Title] OR mental disorder* [Title] OR depress* [tw] OR anxi* [tw] OR affective symptom* [sh] OR mental health condition* [tw] OR mental health [sh] mental ill* [tw] OR mood disorder* [tw] |
| Work context terms | DE "Work (Attitudes Toward)" OR DE "Occupations” OR DE "Occupational Attitudes" OR DE "Occupational Safety" OR DE "Occupational Stress" OR DE "Employment Status" OR DE “Personnel” OR DE “Working Conditions” OR DE “Working Space” OR exp working conditions OR exp organi?ational behavio?r OR exp Organi?ations OR exp Business Organi?ations |
| Work context terms | TI work* OR TI occupation* OR TI employ* OR TI job* OR TI professional* OR TI organi?ational OR work place* [tw] OR workplace* [tw] OR business* [tw] |
| Search for DMHI | (TI(digital OR online OR web-based OR internet OR eHealth OR mHealth OR mobile app OR smartphone OR technology OR computerized OR teletherapy OR virtual) OR AB(digital OR online OR web-based OR internet OR eHealth OR mHealth OR mobile app OR smartphone OR technology OR computerized OR teletherapy OR virtual)) |
| Search for interventions | exp Health Promotion OR exp Stress Management OR occupational intervention* [tw] OR occupational therap* [tw] OR prevent* [tw] OR intervention* [tw] stress manag* [tw] OR program* [tw] OR polic* [tw] OR therap* [tw] OR tool [tw] OR framework [tw] OR support* [tw]  ("cognitive behavioral therapy" OR CBT OR "CBT-based" OR "internet-based CBT" OR "mindfulness" OR "mindfulness-based" OR "MBCT" OR "MBSR" OR "stress management" OR "stress reduction" OR "relaxation techniques" OR "relaxation therapy" OR "positive psychology" OR "self-guided therapy" OR "self-help" OR "problem-solving therapy" OR "acceptance and commitment therapy" OR ACT OR "dialectical behavior therapy" OR DBT OR "behavioral activation" OR "third-wave therapy" OR "mental contrasting" OR "resilience training" OR "emotion regulation training" OR "psychoeducation") |
| Study type | ((DE "Randomized Controlled Trials" OR TI(randomized OR randomised OR RCT) OR AB(randomized OR randomised OR RCT)) |
| Limited | (human and english language and adulthood <18+ years>) |

| Description CENTRAL | AND OR method |
| --- | --- |
| Mental health terms | stress* [Tittle/Abstract] OR mental health [Title] OR mental illness [Title] OR mental disorder* [Title] OR depress* [tw] OR anxi* [tw] OR affective symptom* [sh] OR mental health condition* [tw] OR mental health [sh] mental ill* [tw] OR mood disorder* [tw] |
| Work context terms | DE “Work” OR DE “Work/PF” OR DE “Workload” OR DE “Work environment” OR DE “Occupational Health” OR DE “Occupational Diseases” OR DE “Named Groups by Occupation” OR DE “Occupational Exposure” OR DE “Occupations and Professions” OR DE “Women, Working” OR DE “Employment” OR DE "Burnout, Professional" |
| Work context terms | TI work* OR TI occupation* OR TI employ* OR TI job* OR TI professional* OR TI organi?ational OR work place* [tw] OR workplace* [tw] OR business* [tw] |
| Search for DMHI | (digital OR online OR web-based OR internet OR eHealth OR mHealth OR mobile app OR smartphone OR virtual OR teletherapy OR computerized OR technology-based) |
| Search for interventions | exp Self Care OR occupational intervention* [tw] OR occupational therap* [tw] OR prevent* [tw] OR intervention* [tw] stress manag* [tw] OR program* [tw] OR polic* [tw] OR therap* [tw] OR tool [tw]  ("cognitive behavioral therapy" OR CBT OR "CBT-based" OR "internet-based CBT" OR "mindfulness" OR "mindfulness-based" OR "MBCT" OR "MBSR" OR "stress management" OR "stress reduction" OR "relaxation techniques" OR "relaxation therapy" OR "positive psychology" OR "self-guided therapy" OR "self-help" OR "problem-solving therapy" OR "acceptance and commitment therapy" OR ACT OR "dialectical behavior therapy" OR DBT OR "behavioral activation" OR "third-wave therapy" OR "mental contrasting" OR "resilience training" OR "emotion regulation training" OR "psychoeducation") |
| Study type | ((randomized OR randomised OR RCT OR "controlled trial") |
| Limited | (human and english language and adulthood <18+ years>) |

| Description EMBASE (via Elsevier) | AND OR method |
| --- | --- |
| Mental health terms | stress* [Tittle/Abstract] OR mental health [Title] OR mental illness [Title] OR mental disorder* [Title] OR depress* [tw] OR anxi* [tw] OR affective symptom* [sh] OR mental health condition* [tw] OR mental health [sh] mental ill* [tw] OR mood disorder* [tw] ('depression'/exp OR 'anxiety'/exp OR 'psychological stress'/exp OR 'mental health'/exp OR depression:ti,ab OR anxiety:ti,ab OR stress:ti,ab OR 'common mental disorder':ti,ab OR 'mood disorder':ti,ab) |
| Work context terms | TI work* OR TI occupation* OR TI employ* OR TI job* OR TI professional* OR TI organi?ational OR work place* [tw] OR workplace* [tw] OR business* [tw] ('occupational health'/exp OR 'employment'/exp OR 'workplace'/exp OR workplace:ti,ab OR employee:ti,ab OR employment:ti,ab OR occupational:ti,ab OR job:ti,ab OR organisation*:ti,ab OR worker:ti,ab) |
| Search for DMHI | ('digital health'/exp OR 'mobile application'/exp OR 'internet'/exp OR digital:ti,ab OR online:ti,ab OR 'web-based':ti,ab OR internet:ti,ab OR 'ehealth':ti,ab OR 'mhealth':ti,ab OR 'smartphone':ti,ab OR 'mobile app':ti,ab OR 'technology':ti,ab OR 'teletherapy':ti,ab OR 'computerized':ti,ab OR 'virtual':ti,ab) |
| Search for interventions | ("cognitive behavioral therapy" OR CBT OR "CBT-based" OR "internet-based CBT" OR "mindfulness" OR "mindfulness-based" OR "MBCT" OR "MBSR" OR "stress management" OR "stress reduction" OR "relaxation techniques" OR "relaxation therapy" OR "positive psychology" OR "self-guided therapy" OR "self-help" OR "problem-solving therapy" OR "acceptance and commitment therapy" OR ACT OR "dialectical behavior therapy" OR DBT OR "behavioral activation" OR "third-wave therapy" OR "mental contrasting" OR "resilience training" OR "emotion regulation training" OR "psychoeducation") |
| Study type | ('randomized controlled trial'/exp OR 'randomized controlled trial':ti,ab OR randomised:ti,ab OR randomized:ti,ab OR RCT:ti,ab) |
| Limited | [english]/lim AND [humans]/lim AND [adult]/lim AND [2004–2024]/py |
