# Supplementary material for: Optimizing Workplace Digital Mental Health Interventions: Systematic Review and Meta-Analysis
Source: J Med Internet Res. 2025 Nov 17;27:e71253. doi: 10.2196/71253 (PMC12670063; doi:10.2196/71253)
Supplement: Multimedia Appendix 3 [file jmir_v27i1e71253_app3.docx]

In the Model definition, the observed effect size of each study was the standardized mean difference ($d$) between the intervention group and the control group *post* intervention:

$$d_{\text{post}}=\frac{\mu_{\text{treatment}}-\mu_{\text{control}}}{\sigma_{pooled}}$$

Where:

$$\sigma_{pooled}=\sqrt{\frac{\left( \left( n_{1}-1 \right)\sigma_{1}^{2} \right)+\left( \left( n_{2}-1 \right)\sigma_{2}^{2} \right)}{n_{1}+n_{2}-2}}$$

We accounted for baseline differences between intervention and control arms by including the standardized mean difference between the arms *pre* intervention ($d_{\text{pre}}$) as a covariate-of-no-interest in each model. The resulting adjusted estimates of the therapeutic effect together with its standard error, and are the most precise and least biased estimates of intervention effects according to the Cochrane Statistical Methods Group Deeks et al 2022 *Cochrane Handbook for Systematic Reviews of Interventions*.

To create the moderators of interest, we dummy-coded each independent variable in Table 1, e.g., for *reminder emails*:

$$x=\left\{ \begin{matrix} 0:\text{study A without reminder emails} \\ 1:\text{study B with reminder emails} \end{matrix} \right.$$

Dummy variables for each set of moderators (along with baseline differences and an intercept) were included in the $P\times k$ column matrix ($X_{k}$) for each model.
