# Supplementary figures and images for: Optimizing Workplace Digital Mental Health Interventions: Systematic Review and Meta-Analysis
Source: J Med Internet Res. 2025 Nov 17;27:e71253. doi: 10.2196/71253 (PMC12670063; doi:10.2196/71253)

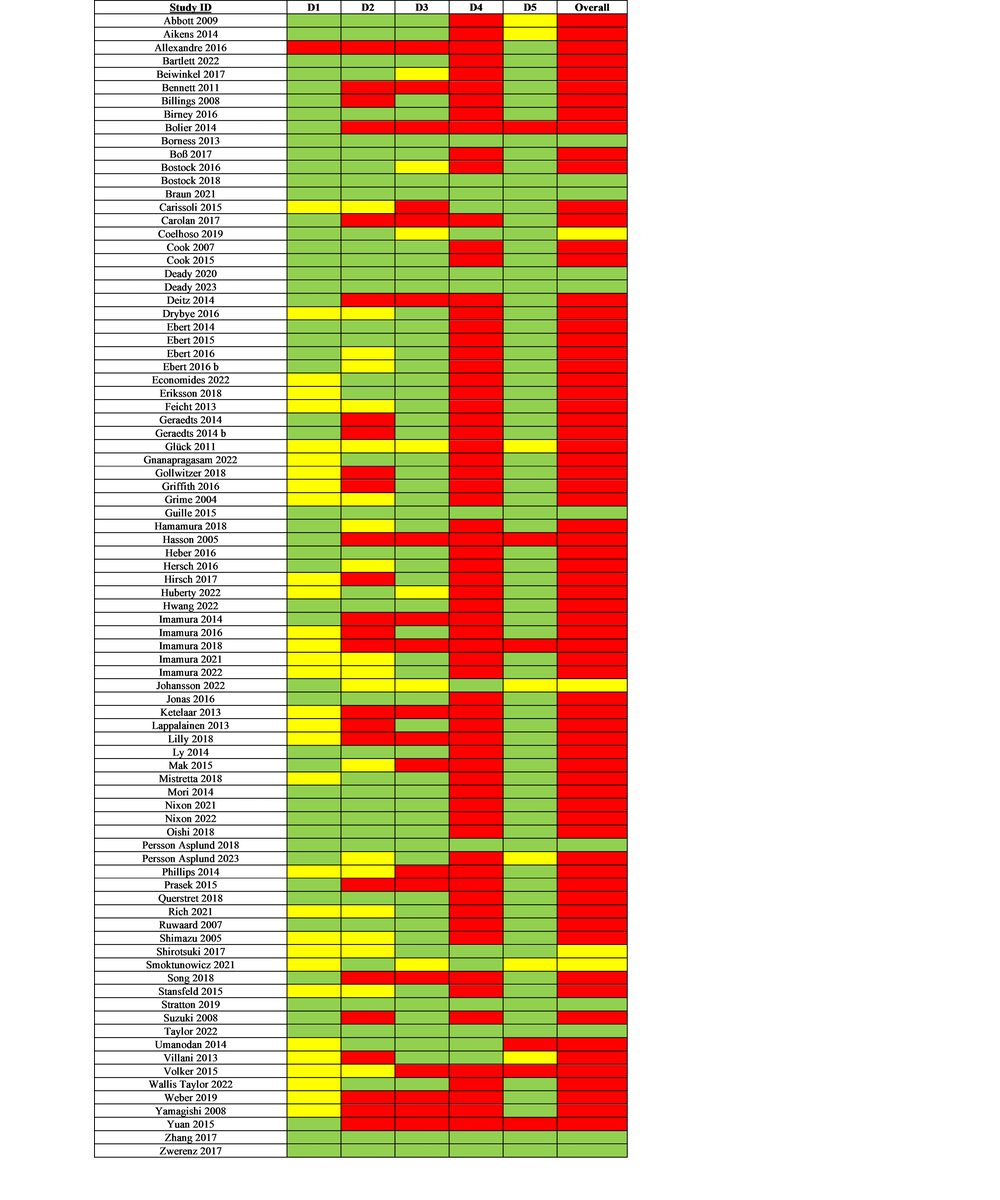

Supplement: Multimedia Appendix 4 [file jmir_v27i1e71253_app4.png]

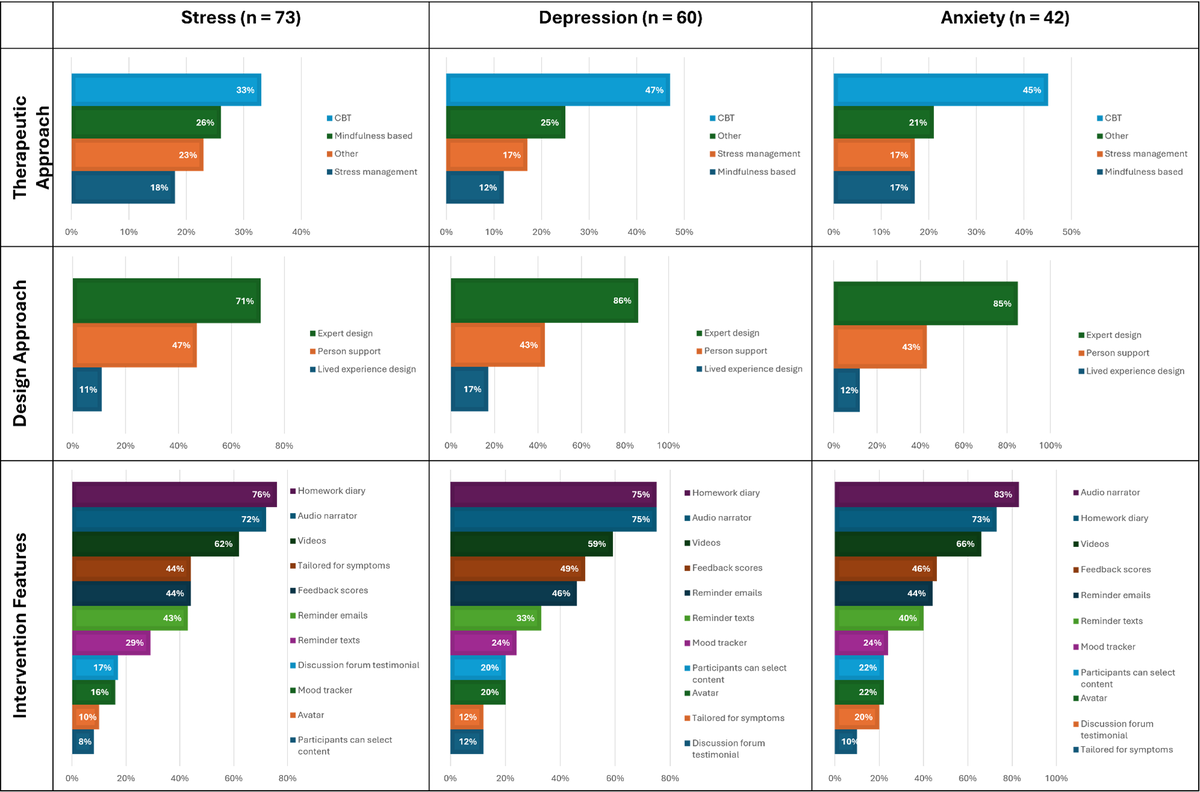

Supplement: Multimedia Appendix 5 [file jmir_v27i1e71253_app5.png]

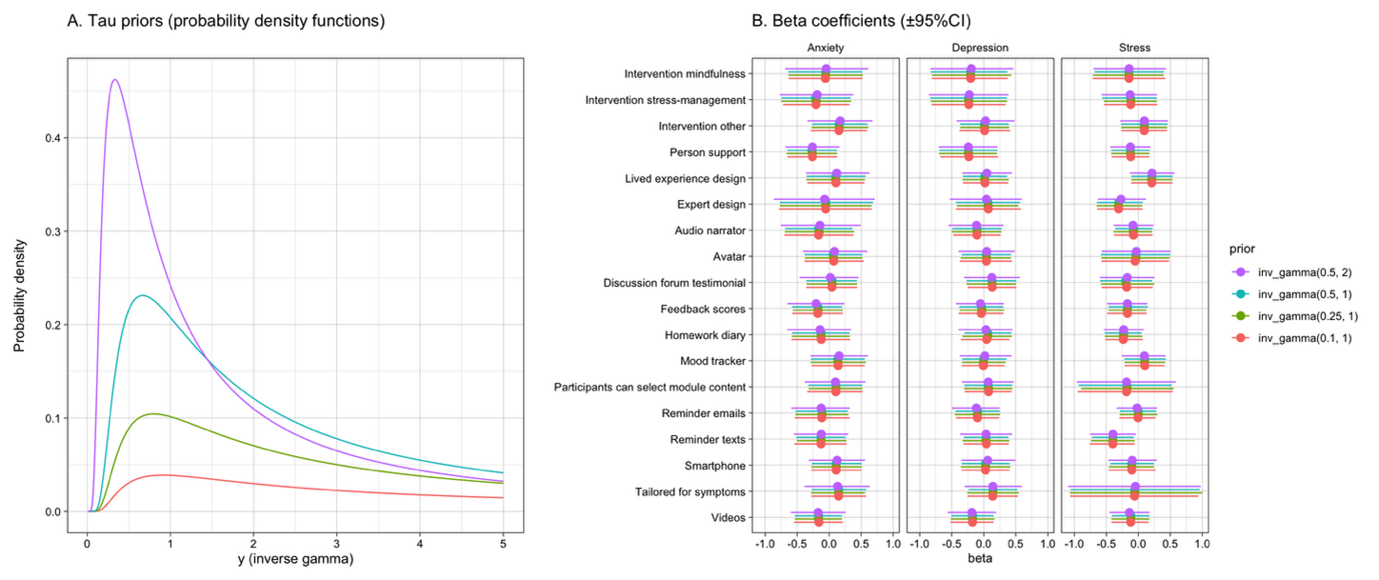

Supplement: Multimedia Appendix 7 [file jmir_v27i1e71253_app7.png]
