# Supplementary material for: Optimizing Workplace Digital Mental Health Interventions: Systematic Review and Meta-Analysis
Source: J Med Internet Res. 2025 Nov 17;27:e71253. doi: 10.2196/71253 (PMC12670063; doi:10.2196/71253)
Supplement: Multimedia Appendix 6 [file jmir_v27i1e71253_app6.docx]

| **Prior** | **Stress** | **Depression** | **Anxiety** |
| --- | --- | --- | --- |
| **inv_gamma(0.50, 1)** | **0.28 [0.19, 0.4]** | **0.2 [0.11, 0.34]** | **0.23 [0.12, 0.38]** |
| **inv_gamma(0.25, 1)** | **0.28 [0.19, 0.4]** | **0.21 [0.11, 0.34]** | **0.23 [0.12, 0.39]** |
| **inv_gamma(0.10, 1)** | **0.28 [0.19, 0.4]** | **0.21 [0.12, 0.35]** | **0.23 [0.13, 0.39]** |
| **inv_gamma(0.50, 2)** | **0.31 [0.22, 0.44]** | **0.27 [0.16, 0.42]** | **0.3 [0.18, 0.47]** |
